# Findings of Epstein-Barr Virus Large B-Cell Lymphoma in a Patient With a History of Rectal Adenocarcinoma: A Case Report

**DOI:** 10.7759/cureus.40680

**Published:** 2023-06-20

**Authors:** Mirna El Dirani, Julius M Nagaratnam, Thebuoshon Amalathasan, Chandni Patel, Mutah Kholoki, Samer Kholoki

**Affiliations:** 1 Internal Medicine, Saint James School of Medicine, Chicago, USA; 2 General Surgery, Avalon University School of Medicine, Phoenix, USA; 3 General Surgery, All Saints University, School of Medicine, Chicago, USA; 4 General Surgery, Saint George's University School of Medicine, Chicago, USA; 5 Internal Medicine, Intermed Limited, Chicago, USA; 6 Internal Medicine, La Grange Memorial Hospital, Chicago, USA

**Keywords:** abdominoperineal resection, colostomy creation, post chemotherapy complication, ebv- positive diffuse large b-cell lymphoma, rectal adenocarcinoma

## Abstract

Colorectal adenocarcinoma is the neoplastic proliferation of glandular tissue in the distal gastrointestinal system and can be managed using surgical resection, novel chemotherapeutic regimens, and radiation therapy. Epstein-Barr virus (EBV) is a common double-stranded DNA virus that has the potential to transform B-cells into lymphoproliferative disorders given the presence of particular conditions such as immunocompromised and chronic inflammatory states. Colorectal cancer is one of the most common malignancies worldwide; however, the additional finding of EBV-positive lymphoma in a patient with a history of colorectal malignancy is uncommon, and this phenomenon has not been thoroughly explored. This report investigates the association between rectal adenocarcinoma and EBV-positive large B-cell lymphoma in an 87-year-old Caucasian male residing in the United States and explores possible causes for this occurrence.

## Introduction

Patients with concerning symptoms such as gastrointestinal bleeding are often referred for a diagnostic colonoscopy to assess for the presence of a colorectal malignancy. Colorectal cancer is the fourth most diagnosed cancer in the world, accounting for 10% of global cancer incidence in 2020 [1,2], and the third leading cause of cancer-related deaths, accounting for 9.4% of cancer mortality in 2020 [1,2]. In 2040, there is a predicted incidence of 3.2 million new colorectal cancer cases [2]. As of 2021, the United States Preventive Services Task Force made changes to the colorectal cancer screening guideline, with the new recommendation of beginning screening at the age of 45 years with subsequent screenings every 10 years until the age of 75 years [3], after which screening is provided on a case-by-case basis until the age of 85 years, where screening is discontinued [3]. The screening guidelines are further altered for patients with a family history of colorectal cancer, or prior positive findings on colonoscopy [3].

Masses and polyps observed during a colonoscopy are often excised or biopsied for pathological evaluation for neoplastic cellular changes and further genetic testing [4]. In most cases, two predominant genetic pathways are involved in the pathogenesis of colorectal carcinoma: the chromosomal instability (CIN) pathway and the mutator pathway [4,5]. The mutator pathway involves the mutation of mismatch genes such as MSH-2, MSH-6, and MLH-1, which often results in conditions such as Lynch syndrome [5,6]. The CIN pathway in contrast involves the mutation of *APC*, *K-RAS*, and *TP53* genes, leading to the neoplastic transformation of colorectal tissue [5,6]. Following genetic testing, the focus turns to the use of an appropriate chemotherapeutic combination to manage the patient’s colorectal malignancy. A commonly used chemotherapeutic combination is the FOLFOX regimen, which consists of Leucovorin or (FOL)inic Acid, 5-(F)luorouracial, and (OX)platin [7], which was approved in the United States after promising results were observed in the 2004 Multicenter International Study of Oxaliplatin/5-Fluorouracil/Leucovorin in the Adjuvant Treatment of Colon Cancer (MOSAIC) trial [7].

After chemotherapy, patients become immunocompromised and are susceptible to infection and recurrence of malignancy [8]. A commonly transmitted infectious source in immunocompromised patients is the oncovirus Epstein-Barr virus (EBV) [9]. EBV is commonly associated with the development of lymphoproliferative disorders such as large B-cell lymphoma, Burkitt’s lymphoma, and Hodgkin’s lymphoma [9]. To date, there have been very few documented cases of developing EBV-positive lymphoproliferative disorders in a patient with a history of colorectal malignancy, and hence further evaluation of the pathophysiology is required to understand this phenomenon.

## Case presentation

On May 28, 2021, an 87-year-old Caucasian male presented for a scheduled outpatient colonoscopy. The procedure was scheduled due to the patient’s complaints of blood and mucus in his stool, and increasingly soft consistency of his stool over the past few months. The patient has a history of regular colonoscopies, with his previous positive colonoscopy being in 2007, which involved the removal of 15 colonic polyps, none of which were ruled as being malignant. The patient also has a past medical history significant for atrial fibrillation with slow ventricular rate (on warfarin therapy for stroke prevention), coronary artery disease, hypertension, hyperlipidemia, obstructive sleep apnea, gouty arthritis, malignant melanoma of the forehead and squamous cell carcinoma of the nose both requiring resection and grafting, and prostate cancer requiring brachytherapy and subsequent prostatectomy. The patient was also a former smoker who quit in 1990.

The colonoscopy showed a hard, ulcerated circumferential mid rectal mass in the rectal ampulla. The mass had bloody discharge and appeared consistent with primary rectal cancer. The mass was biopsied multiple times, and then the colonoscope was passed further up where further 19 polyps were removed using a cold snare technique; a 0.9cm x 0.25cm x 0.2cm polyp was removed from the distal ascending colon, five proximal transverse colon polyps aggregating at 1.6cm x 1.0cm x 0.3cm in size were removed, four mid transverse colon polyps ranging between 0.6cm and 0.9cm were removed, five proximal descending colon polyps ranging between 0.5cm and 1.0cm were removed, a mid-descending colon polyp measuring 1.1cm x 0.9cm x 0.3cm was removed, and two distal sigmoid polyps were removed, one of which had a 0.4cm mass attached to a 0.7cm pedicle and the second being 0.8cm x 0.6cm x 0.5cm in size and required a clip placement after excision due to excessive bleeding. A 0.9cm x 0.7cm x 0.2cm mass was then located on the ileocecal junction, which was adenomatous in nature after histological evaluation. Due to the size and location of the mass, it could not be removed and was biopsied. Finally, a 1cm x 0.15cm x 0.1cm polyp on the cecum was removed by cold biopsy. After the procedure, the patient was advised to hold his warfarin regimen for five days to observe for blood in the stool, and if there was an absence of blood, then the patient was permitted to resume his regimen.

The pathology results showed that the cecal polyp contained fragments of colonic mucosa with focal lymphoid aggregates. The distal ascending, transverse, mid descending, three out of five proximal descending, and the distal sigmoid colon polyps were found to be tubular adenomas. The remaining two proximal descending polyps were found to be fragments of colonic mucosa with no significant diagnostic abnormalities. The mid rectal mass biopsy was found to be an invasive well-differentiated adenocarcinoma arising in a background of tubulovillous adenoma. Based on the pathology findings, the patient was referred to surgery for consideration for an ileocolectomy to remove the ileocecal valve mass and abdominoperineal resection (APR) versus minimally invasive surgery on the ileocecal valve with subsequent radiation of the rectum.

**Figure 1:**
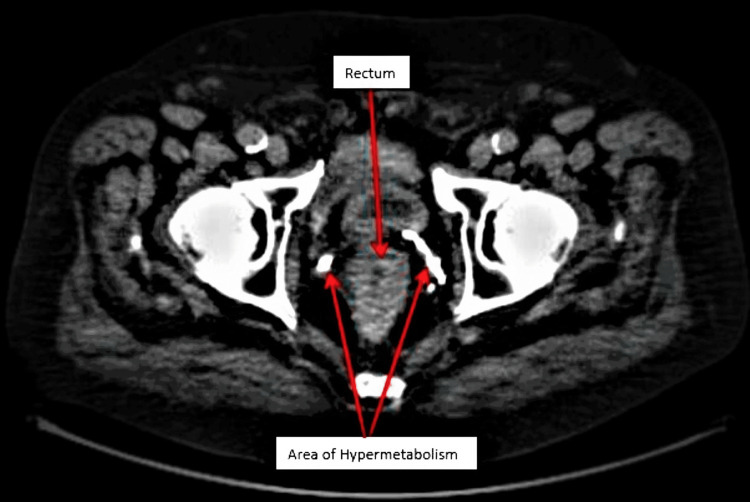
PET scan showing area of hypermetabolism in the perirectal region. PET, positron emission tomography

On July 12, 2021, the patient underwent an endoscopic ultrasound (EUS) to stage his newly diagnosed rectal cancer. EUS findings showed a mass invading the muscle wall layer and into the adventitia, consistent with a T3 staging, and the presence of one malignant appearing lymph node measuring 9mm in size, giving the final diagnosis of a T3N1 rectal adenocarcinoma. After the EUS findings, the patient was referred to oncology for chemotherapeutic and possible radiation therapy. A positron emission tomography (PET) scan was performed on July 30, 2021, to assess for the extent of the metastasis of the rectal adenocarcinoma. The PET scan findings showed the presence of rectal hypermetabolism extending a length of 5.7cm, with a peak standardized uptake value (SUV) of 11.3cm, without any hypermetabolic lymphadenopathy (Figure 1). The patient was then started on a neoadjuvant chemoradiation therapy prior to surgical intervention.

The patient presented for an elective surgery on December 6, 2021, for the management of his rectal cancer and mass on the ileocecal valve. The patient underwent a laparoscopic-assisted APR with omental pedicle flap and colostomy creation, lymphonodectomy of three superior hemorrhoidal lymph nodes, and administration of a transversus abdominis plane block using 60mL of 0.25% ropivacaine. The procedure was complicated with an unavoidable 2mm dissection of the bladder requiring repair with two layers of vicryl sutures. The error was ruled unavoidable due to the patient’s history of prostatectomy, which caused the bladder to be distended and obstructing the view. The location of the tumor was another reason behind the unavoidable dissection. Two tissue samples of the bladder margin (1.7cm x 1.3cm x 1.5cm and 1.4cm x 0.7cm x 0.5cm) were also sent to surgical pathology and returned back negative for the tumor. The superior hemorrhoidal lymph nodes, aggregating at 11.6cm x 9.2cm x 4.3cm, were negative for metastatic carcinoma. There was no other evidence of metastatic disease observed within the abdomen during the procedure.

The 5.3cm rectal mass obtained from the laparoscopic-assisted APR showed residual well-differentiated adenocarcinoma associated with tubulovillous adenoma, with tumor invasion through the muscularis propria into the pericolorectal soft tissues, and all margins being negative of invasive carcinoma (the closest circumferential margin being 5mm from the carcinoma). There was a near-complete mesorectal envelope, with one out of 27 lymph nodes showing isolated tumor cells associated with acellular mucin pools. After a cystogram was performed, which showed no evidence of bladder leakage post-dissection repair, the patient was discharged on December 13, 2021.

The surgical samples were then sent for further immunohistochemistry staining and genetic study through ordering a RAS/PAF panel and HER-2 colorectal immunohistochemistry. On March 21, 2022, the samples were positive for KRAS with the presence of a 20.0% variant allele frequency secondary to a missense mutation. The samples were negative for HER-2, BRAF, HRAS, AND NRAS. Per the chemotherapeutic guidelines, the patient was not a candidate for an anti-EGFR chemotherapy regimen such as cetuximab and panitumumab due to the neoplasms lack of response to the regimen, and the patient was started on an adjuvant capecitabine therapy, which was completed in June 2022.

**Figure 2:**
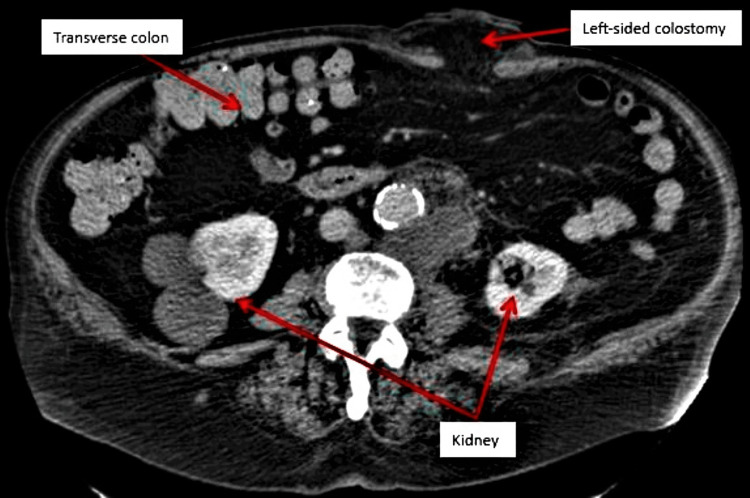
Abdominal CT after APR, colostomy creation, and chemotherapy CT, computed tomography; APR, abdominoperineal resection

A computed tomography (CT) scan of the chest, abdomen, and pelvis with contrast was performed on July 6, 2022, to assess again for metastatic disease after completion of his chemotherapy. The CT scan showed a left-sided colostomy site with mild-to-moderate parastomal herniation of mesenteric fat (Figure 2) and no evidence of metastatic disease in the abdomen and chest.

On December 3, 2022, the patient was admitted to the emergency department for diffuse lower abdominal pain and lack of output from his colostomy for four days. The patient also had diminished oral intake during the period. The patient also complained of left-sided chest pain for the past two days and denied any symptoms of fever, melena, hematemesis, nausea, or vomiting. Physical examination showed lymphadenopathy in the right occipital (3 x 2cm) and cervical region, left supraclavicular region, and the mid-abdominal region. CT of the abdomen and pelvis with contrast showed extensive retroperitoneal lymphadenopathy compressing the left renal vein and the presence of a right renal lesion suspicious for metastatic disease. CT of the chest and neck with contrast showed no evidence of an acute pulmonary embolism; however, it showed a left enlarged supraclavicular lymph node (3.8cm x 2.7cm) and mediastinal lymphadenopathy involving the superior and anterior mediastinum. Additional lymphadenopathy was observed in the right paratracheal region, prevascular, paraesophageal, subcarinal, retrocrural, peripancreatic, periportal, gastrohepatic, and retroperitoneal regions. Ultrasound-guided fine needle biopsy and core biopsy were performed on the left supraclavicular lymph nodes, with the results of the fine needle biopsy showing an EBV-positive lymphoma with extensive necrosis, favoring a large B-cell lymphoma. The patient started having improved colostomy output with the administration of colace 100mg twice daily (BID), and the patient was discharged on December 8, 2022. The patient's EBV-positive lymphoma was managed with the chemotherapeutic R-mini-CHOP regimen starting March 3, 2023, comprising of (R)ituximab, (C)yclophosphamide, (H)ydroxydaunorubicin, (O)ncovin or vincristine, and (P)rednisone.

## Discussion

The rise of EBV-positive lymphoma in our patient with a recent history of rectal adenocarcinoma has led us to ask intriguing questions about the possible associations and the interplay between these two malignancies. While our patient's EBV infection was only discovered after the resolution of the adenocarcinoma, the prospect still exists that the EBV infection was co-present during the time of malignancy. The probability also exists that the nature of the adenocarcinoma and its prescribed treatment could have resulted in the reactivation or increased susceptibility to EBV infection.

Firstly, the findings of EBV-positive lymphoma in our patient with a history of rectal adenocarcinoma suggest a potential relationship between the two malignancies. While the link may not be direct, it could indicate shared underlying factors or pathways that contribute to their development, as explored in other studies. A prior study has demonstrated the presence of EBV infection in rectal adenocarcinoma cases using in situ hybridization for EBV-encoded small RNAs [10]. This finding suggests that EBV infection may play a role in the development or progression of a subset of rectal adenocarcinomas. A study conducted in 2010 identified shared pathways between EBV-positive lymphoma and colorectal cancer [11], such as the activation of the Wnt/β-catenin signaling pathway by EBV-encoded latent membrane protein 1 (LMP1), which is also implicated in colorectal cancer development [11]. There have been findings that show the impact of immune evasion by EBV on tumor surveillance [12], which may have played a part in concealing the EBV lymphoma while concurrently expressing the rectal adenocarcinoma found in our patient [12]. These findings suggest potential common pathways and immune dysregulation mechanisms that could contribute to the development of both rectal adenocarcinoma and EBV-positive lymphoma.

Secondly, the chronic inflammatory state created by malignancies such as rectal adenocarcinoma may provide an ideal environment for acquiring an EBV infection, which can subsequently progress to a lymphoma. Involvement of proinflammatory proteins such as annexin A1 and FPR2/ALX aid in the progressive proliferation and eventual metastasis of rectal adenocarcinoma [13]. Chronic inflammatory states commonly have increased levels of NF-κB, which are also present in the setting of gastrointestinal malignancies [14]. NF-κB is essential for the survival and proliferation of Hodgkin Reed Sternberg cells, a common malignancy secondary to EBV [14,15]. EBV-positive large B-cell proliferations have been observed in sites of malignancy, which are now classified as diffuse large B-cell lymphoma associated with chronic inflammation (DLBCL-CI) in accordance with the World Health Organization classification [16].

Lastly, the chemotherapy used for treating rectal adenocarcinoma, specifically adjuvant capecitabine, can suppress the immune system’s ability to control viral infections [8]. EBV, an oncovirus responsible for lymphoproliferative disorders, is particularly associated with individuals with immunosuppression. Studies that analyzed the effects of adjuvant chemotherapy on pediatric patients receiving bone marrow transplant as a method of preventing rejection were susceptible to EBV infection due to immunosuppressive properties of the treatment [17]. Our patient's immunosuppressive state after the effects of adjuvant chemotherapy may have contributed to the reactivation of latent EBV infection or increased the patient's susceptibility to an EBV infection, ultimately leading to the development of EBV-positive lymphoma. Further examination of the impact of chemotherapy on immune function and viral susceptibility could provide insight into the methods of preventing development of secondary malignancies such as EBV-positive lymphoma.

## Conclusions

The relationship between rectal adenocarcinoma and EBV-positive lymphoma, as explored in this case, is complex and contains many underlying molecular and physiological pathways. This case shows that there are multiple possible explanations for the findings of EBV-positive lymphoma in a patient with a recent history of rectal adenocarcinoma. There will be some clinical benefits behind advocating for large-scale studies to further assess this relationship and hence determine the most plausible causes of this phenomenon. Conducting large-scale studies can be also useful in determining preventive therapies for the development of EBV infection and progression to malignancy.
